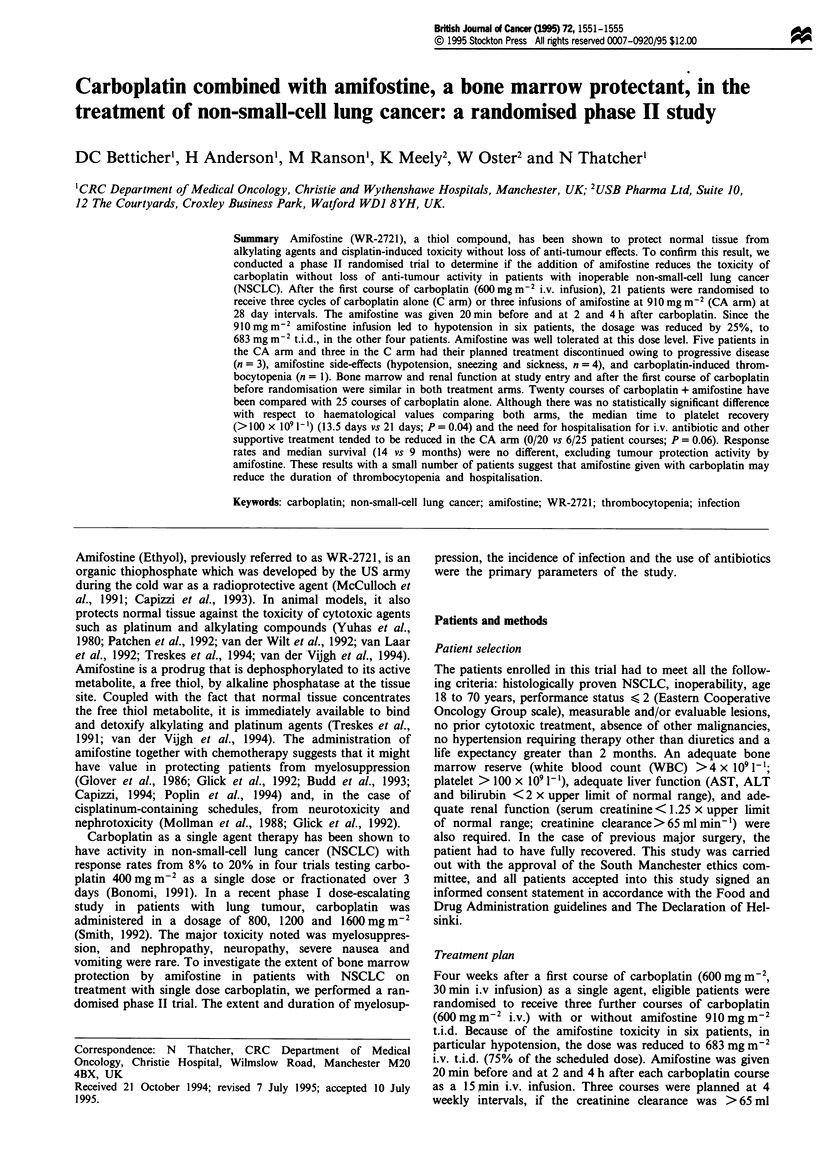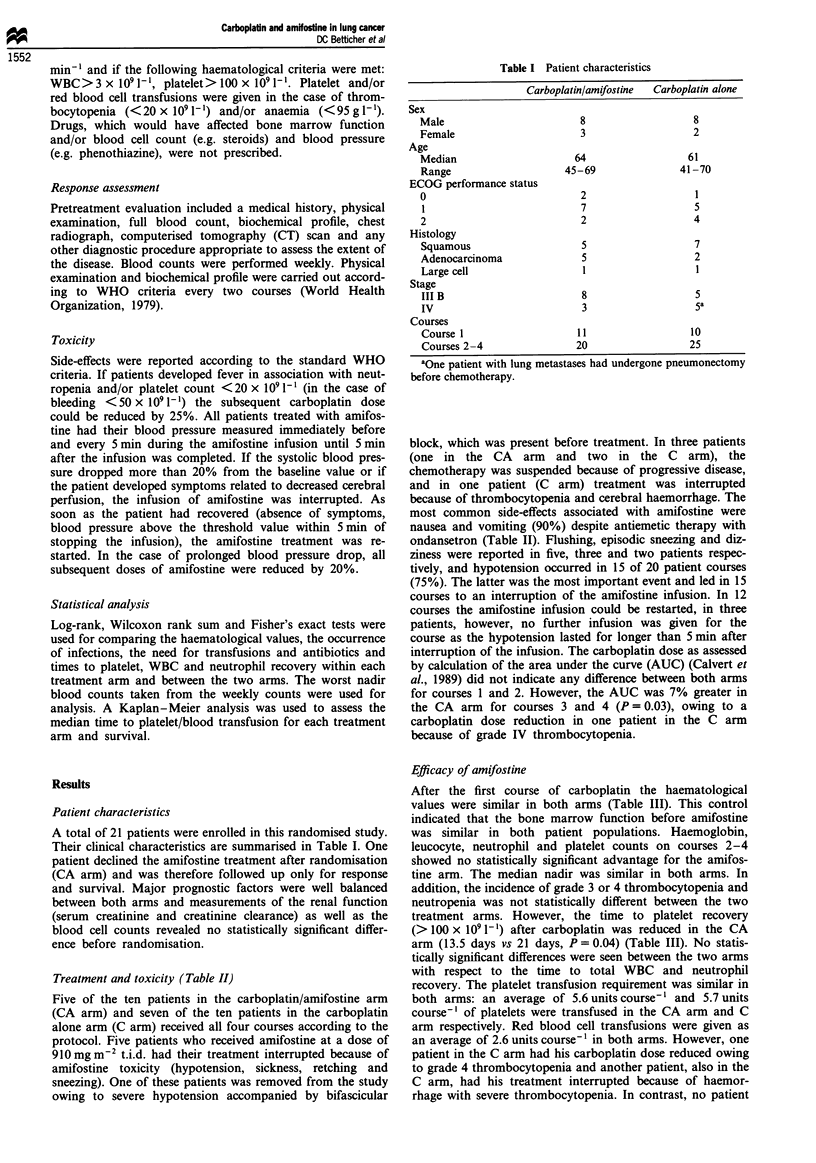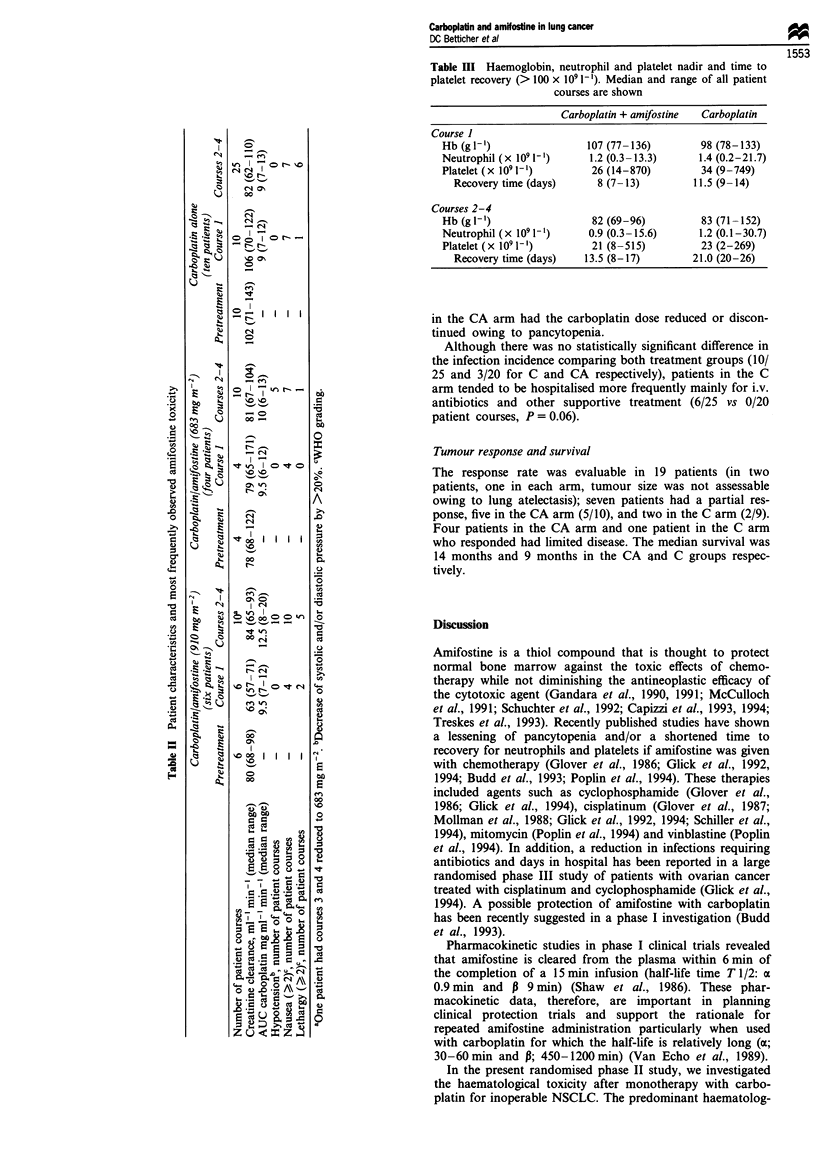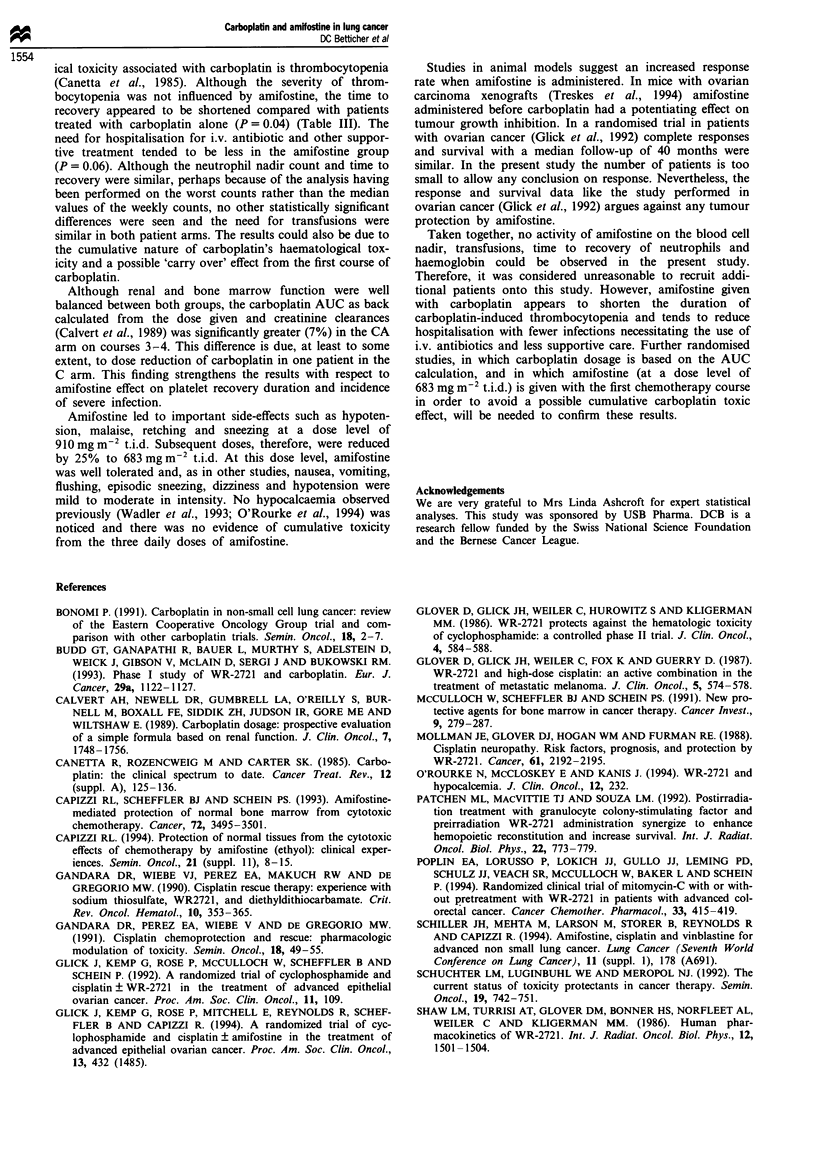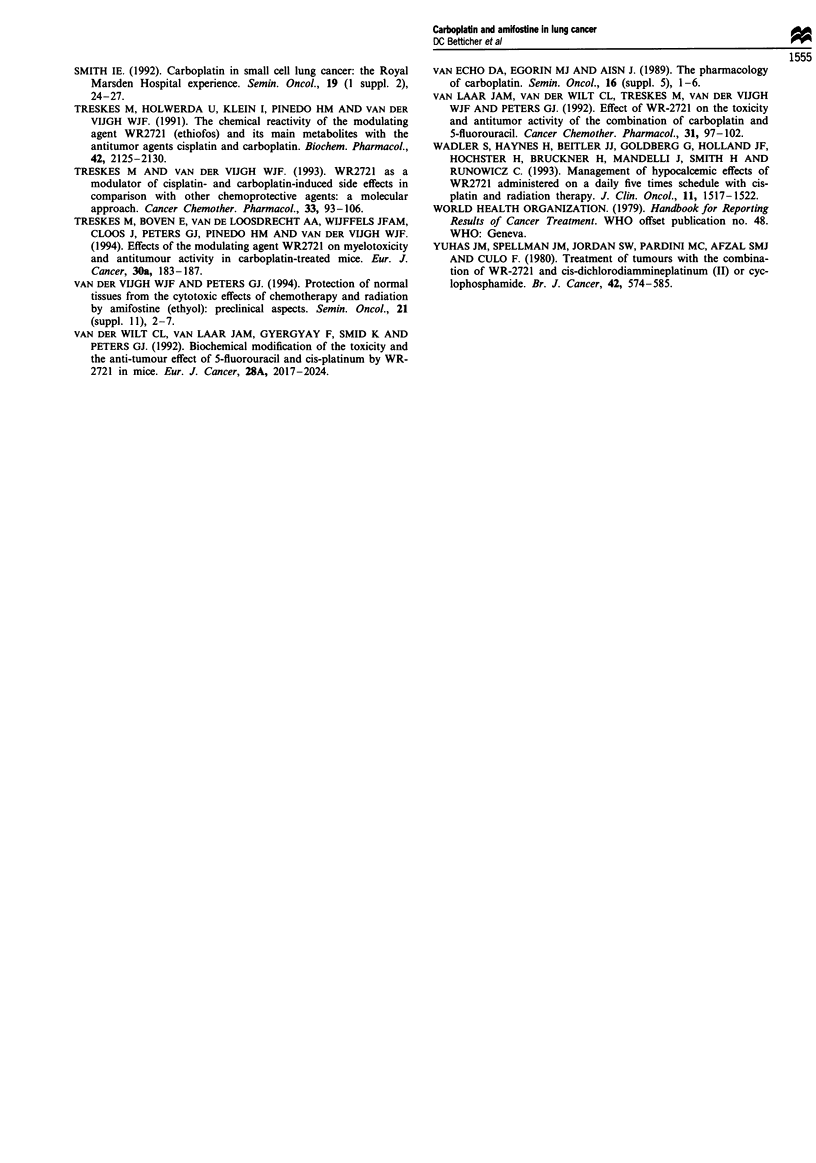# Carboplatin combined with amifostine, a bone marrow protectant, in the treatment of non-small-cell lung cancer: a randomised phase II study.

**DOI:** 10.1038/bjc.1995.546

**Published:** 1995-12

**Authors:** D. C. Betticher, H. Anderson, M. Ranson, K. Meely, W. Oster, N. Thatcher

**Affiliations:** CRC Department of Medical Oncology, Christie and Wythenshawe Hospitals, Manchester, UK.

## Abstract

Amifostine (WR-2721), a thiol compound, has been shown to protect normal tissue from alkylating agents and cisplatin-induced toxicity without loss of anti-tumour effects. To confirm this result, we conducted a phase II randomised trial to determine if the addition of amifostine reduces the toxicity of carboplatin without loss of anti-tumour activity in patients with inoperable non-small-cell lung cancer (NSCLC). After the first course of carboplatin (600 mg m-2 i.v. infusion), 21 patients were randomised to receive three cycles of carboplatin alone (C arm) or three infusions of amifostine at 910 mg m-2 (CA arm) at 28 day intervals. The amifostine was given 20 min before and at 2 and 4 h after carboplatin. Since the 910 mg m-2 amifostine infusion led to hypotension in six patients, the dosage was reduced by 25%, to 683 mg m-2 t.i.d., in the other four patients. Amifostine was well tolerated at this dose level. Five patients in the CA arm and three in the C arm had their planned treatment discontinued owing to progressive disease (n = 3), amifostine side-effects (hypotension, sneezing and sickness, n = 4), and carboplatin-induced thrombocytopenia (n = 1). Bone marrow and renal function at study entry and after the first course of carboplatin before randomisation were similar in both treatment arms. Twenty courses of carboplatin+amifostine have been compared with 25 courses of carboplatin alone. Although there was no statistically significant difference with respect to haematological values comparing both arms, the median time to platelet recovery (> 100 x 10(9) l-1) (13.5 days vs 21 days; P = 0.04) and the need for hospitalisation for i.v. antibiotic and other supportive treatment tended to be reduced in the CA arm (0/20 vs 6/25 patient courses; P = 0.06). Response rates and median survival (14 vs 9 months) were no different, excluding tumour protection activity by amifostine. These results with a small number of patients suggest that amifostine given with carboplatin may reduce the duration of thrombocytopenia and hospitalisation.


British Journal of Cancer (1995) 72, 1551–1555
© 1995 Stockton Press All rights reserved 0007-0920/95 $12.00

### Carboplatin combined with amifostine, a bone marrow protectant, in the treatment of non-small-cell lung cancer: a randomised phase II study

DC Betticher[1], H Anderson[1], M Ranson[1], K Meely[2], W Oster[2] and N Thatcher[1]

[1]*CRC Department of Medical Oncology, Christie and Wythenshawe Hospitals, Manchester, UK;* [2]*USB Pharma Ltd, Suite 10, 12 The Courtyards, Croxley Business Park, Watford WD1 8YH, UK.*

**Summary** Amifostine (WR-2721), a thiol compound, has been shown to protect normal tissue from alkylating agents and cisplatin-induced toxicity without loss of anti-tumour effects. To confirm this result, we conducted a phase II randomised trial to determine if the addition of amifostine reduces the toxicity of carboplatin without loss of anti-tumour activity in patients with inoperable non-small-cell lung cancer (NSCLC). After the first course of carboplatin (600 mg $m^{-2}$ i.v. infusion), 21 patients were randomised to receive three cycles of carboplatin alone (C arm) or three infusions of amifostine at 910 mg $m^{-2}$ (CA arm) at 28 day intervals. The amifostine was given 20 min before and at 2 and 4 h after carboplatin. Since the 910 mg $m^{-2}$ amifostine infusion led to hypotension in six patients, the dosage was reduced by 25%, to 683 mg $m^{-2}$ t.i.d., in the other four patients. Amifostine was well tolerated at this dose level. Five patients in the CA arm and three in the C arm had their planned treatment discontinued owing to progressive disease ($n = 3$), amifostine side-effects (hypotension, sneezing and sickness, $n = 4$), and carboplatin-induced thrombocytopenia ($n = 1$). Bone marrow and renal function at study entry and after the first course of carboplatin before randomisation were similar in both treatment arms. Twenty courses of carboplatin + amifostine have been compared with 25 courses of carboplatin alone. Although there was no statistically significant difference with respect to haematological values comparing both arms, the median time to platelet recovery ($>100 \times 10^9 l^{-1}$) (13.5 days *vs* 21 days; $P = 0.04$) and the need for hospitalisation for i.v. antibiotic and other supportive treatment tended to be reduced in the CA arm (0/20 *vs* 6/25 patient courses; $P = 0.06$). Response rates and median survival (14 *vs* 9 months) were no different, excluding tumour protection activity by amifostine. These results with a small number of patients suggest that amifostine given with carboplatin may reduce the duration of thrombocytopenia and hospitalisation.

**Keywords**: carboplatin; non-small-cell lung cancer; amifostine; WR-2721; thrombocytopenia; infection

Amifostine (Ethyol), previously referred to as WR-2721, is an organic thiophosphate which was developed by the US army during the cold war as a radioprotective agent (McCulloch *et al.*, 1991; Capizzi *et al.*, 1993). In animal models, it also protects normal tissue against the toxicity of cytotoxic agents such as platinum and alkylating compounds (Yuhas *et al.*, 1980; Patchen *et al.*, 1992; van der Wilt *et al.*, 1992; van Laar *et al.*, 1992; Treskes *et al.*, 1994; van der Vijgh *et al.*, 1994). Amifostine is a prodrug that is dephosphorylated to its active metabolite, a free thiol, by alkaline phosphatase at the tissue site. Coupled with the fact that normal tissue concentrates the free thiol metabolite, it is immediately available to bind and detoxify alkylating and platinum agents (Treskes *et al.*, 1991; van der Vijgh *et al.*, 1994). The administration of amifostine together with chemotherapy suggests that it might have value in protecting patients from myelosuppression (Glover *et al.*, 1986; Glick *et al.*, 1992; Budd *et al.*, 1993; Capizzi, 1994; Poplin *et al.*, 1994) and, in the case of cisplatinum-containing schedules, from neurotoxicity and nephrotoxicity (Mollman *et al.*, 1988; Glick *et al.*, 1992).

Carboplatin as a single agent therapy has been shown to have activity in non-small-cell lung cancer (NSCLC) with response rates from 8% to 20% in four trials testing carboplatin 400 mg $m^{-2}$ as a single dose or fractionated over 3 days (Bonomi, 1991). In a recent phase I dose-escalating study in patients with lung tumour, carboplatin was administered in a dosage of 800, 1200 and 1600 mg $m^{-2}$ (Smith, 1992). The major toxicity noted was myelosuppression, and nephropathy, neuropathy, severe nausea and vomiting were rare. To investigate the extent of bone marrow protection by amifostine in patients with NSCLC on treatment with single dose carboplatin, we performed a randomised phase II trial. The extent and duration of myelosuppression, the incidence of infection and the use of antibiotics were the primary parameters of the study.

Correspondence: N Thatcher, CRC Department of Medical Oncology, Christie Hospital, Wilmslow Road, Manchester M20 4BX, UK
Received 21 October 1994; revised 7 July 1995; accepted 10 July 1995.

### Patients and methods

### *Patient selection*

The patients enrolled in this trial had to meet all the following criteria: histologically proven NSCLC, inoperability, age 18 to 70 years, performance status $\leqslant 2$ (Eastern Cooperative Oncology Group scale), measurable and/or evaluable lesions, no prior cytotoxic treatment, absence of other malignancies, no hypertension requiring therapy other than diuretics and a life expectancy greater than 2 months. An adequate bone marrow reserve (white blood count (WBC) $>4 \times 10^9 l^{-1}$; platelet $>100 \times 10^9 l^{-1}$), adequate liver function (AST, ALT and bilirubin $<2 \times$ upper limit of normal range), and adequate renal function (serum creatinine $<1.25 \times$ upper limit of normal range; creatinine clearance $>65$ ml $min^{-1}$) were also required. In the case of previous major surgery, the patient had to have fully recovered. This study was carried out with the approval of the South Manchester ethics committee, and all patients accepted into this study signed an informed consent statement in accordance with the Food and Drug Administration guidelines and The Declaration of Helsinki.

### *Treatment plan*

Four weeks after a first course of carboplatin (600 mg $m^{-2}$, 30 min i.v infusion) as a single agent, eligible patients were randomised to receive three further courses of carboplatin (600 mg $m^{-2}$ i.v.) with or without amifostine 910 mg $m^{-2}$ t.i.d. Because of the amifostine toxicity in six patients, in particular hypotension, the dose was reduced to 683 mg $m^{-2}$ i.v. t.i.d. (75% of the scheduled dose). Amifostine was given 20 min before and at 2 and 4 h after each carboplatin course as a 15 min i.v. infusion. Three courses were planned at 4 weekly intervals, if the creatinine clearance was $>65$ ml min$^{-1}$ and if the following haematological criteria were met: WBC > $3 \times 10^9 l^{-1}$, platelet > $100 \times 10^9 l^{-1}$. Platelet and/or red blood cell transfusions were given in the case of thrombocytopenia (< $20 \times 10^9 l^{-1}$) and/or anaemia (< $95 g l^{-1}$). Drugs, which would have affected bone marrow function and/or blood cell count (e.g. steroids) and blood pressure (e.g. phenothiazine), were not prescribed.

### *Response assessment*

Pretreatment evaluation included a medical history, physical examination, full blood count, biochemical profile, chest radiograph, computerised tomography (CT) scan and any other diagnostic procedure appropriate to assess the extent of the disease. Blood counts were performed weekly. Physical examination and biochemical profile were carried out according to WHO criteria every two courses (World Health Organization, 1979).

### *Toxicity*

Side-effects were reported according to the standard WHO criteria. If patients developed fever in association with neutropenia and/or platelet count < $20 \times 10^9 l^{-1}$ (in the case of bleeding < $50 \times 10^9 l^{-1}$) the subsequent carboplatin dose could be reduced by 25%. All patients treated with amifostine had their blood pressure measured immediately before and every 5 min during the amifostine infusion until 5 min after the infusion was completed. If the systolic blood pressure dropped more than 20% from the baseline value or if the patient developed symptoms related to decreased cerebral perfusion, the infusion of amifostine was interrupted. As soon as the patient had recovered (absence of symptoms, blood pressure above the threshold value within 5 min of stopping the infusion), the amifostine treatment was restarted. In the case of prolonged blood pressure drop, all subsequent doses of amifostine were reduced by 20%.

### *Statistical analysis*

Log-rank, Wilcoxon rank sum and Fisher's exact tests were used for comparing the haematological values, the occurrence of infections, the need for transfusions and antibiotics and times to platelet, WBC and neutrophil recovery within each treatment arm and between the two arms. The worst nadir blood counts taken from the weekly counts were used for analysis. A Kaplan–Meier analysis was used to assess the median time to platelet/blood transfusion for each treatment arm and survival.

### Results

### *Patient characteristics*

A total of 21 patients were enrolled in this randomised study. Their clinical characteristics are summarised in Table I. One patient declined the amifostine treatment after randomisation (CA arm) and was therefore followed up only for response and survival. Major prognostic factors were well balanced between both arms and measurements of the renal function (serum creatinine and creatinine clearance) as well as the blood cell counts revealed no statistically significant difference before randomisation.

### *Treatment and toxicity (Table II)*

Five of the ten patients in the carboplatin/amifostine arm (CA arm) and seven of the ten patients in the carboplatin alone arm (C arm) received all four courses according to the protocol. Five patients who received amifostine at a dose of 910 mg m$^{-2}$ t.i.d. had their treatment interrupted because of amifostine toxicity (hypotension, sickness, retching and sneezing). One of these patients was removed from the study owing to severe hypotension accompanied by bifascicular block, which was present before treatment. In three patients (one in the CA arm and two in the C arm), the chemotherapy was suspended because of progressive disease, and in one patient (C arm) treatment was interrupted because of thrombocytopenia and cerebral haemorrhage. The most common side-effects associated with amifostine were nausea and vomiting (90%) despite antiemetic therapy with ondansetron (Table II). Flushing, episodic sneezing and dizziness were reported in five, three and two patients respectively, and hypotension occurred in 15 of 20 patient courses (75%). The latter was the most important event and led in 15 courses to an interruption of the amifostine infusion. In 12 courses the amifostine infusion could be restarted, in three patients, however, no further infusion was given for the course as the hypotension lasted for longer than 5 min after interruption of the infusion. The carboplatin dose as assessed by calculation of the area under the curve (AUC) (Calvert *et al.*, 1989) did not indicate any difference between both arms for courses 1 and 2. However, the AUC was 7% greater in the CA arm for courses 3 and 4 ($P = 0.03$), owing to a carboplatin dose reduction in one patient in the C arm because of grade IV thrombocytopenia.

**Table I** Patient characteristics

| | *Carboplatin/amifostine* | *Carboplatin alone* |
|---|---|---|
| Sex | | |
| Male | 8 | 8 |
| Female | 3 | 2 |
| Age | | |
| Median | 64 | 61 |
| Range | 45–69 | 41–70 |
| ECOG performance status | | |
| 0 | 2 | 1 |
| 1 | 7 | 5 |
| 2 | 2 | 4 |
| Histology | | |
| Squamous | 5 | 7 |
| Adenocarcinoma | 5 | 2 |
| Large cell | 1 | 1 |
| Stage | | |
| III B | 8 | 5 |
| IV | 3 | 5[a] |
| Courses | | |
| Course 1 | 11 | 10 |
| Courses 2–4 | 20 | 25 |

[a]One patient with lung metastases had undergone pneumonectomy before chemotherapy.

### *Efficacy of amifostine*

After the first course of carboplatin the haematological values were similar in both arms (Table III). This control indicated that the bone marrow function before amifostine was similar in both patient populations. Haemoglobin, leucocyte, neutrophil and platelet counts on courses 2–4 showed no statistically significant advantage for the amifostine arm. The median nadir was similar in both arms. In addition, the incidence of grade 3 or 4 thrombocytopenia and neutropenia was not statistically different between the two treatment arms. However, the time to platelet recovery (> $100 \times 10^9 l^{-1}$) after carboplatin was reduced in the CA arm (13.5 days *vs* 21 days, $P = 0.04$) (Table III). No statistically significant differences were seen between the two arms with respect to the time to total WBC and neutrophil recovery. The platelet transfusion requirement was similar in both arms: an average of 5.6 units course$^{-1}$ and 5.7 units course$^{-1}$ of platelets were transfused in the CA arm and C arm respectively. Red blood cell transfusions were given as an average of 2.6 units course$^{-1}$ in both arms. However, one patient in the C arm had his carboplatin dose reduced owing to grade 4 thrombocytopenia and another patient, also in the C arm, had his treatment interrupted because of haemorrhage with severe thrombocytopenia. In contrast, no patient

**Table II** Patient characteristics and most frequently observed amifostine toxicity

| | *Carboplatin/amifostine (910 mg m⁻²) (six patients)* | | | *Carboplatin/amifostine (683 mg m⁻²) (four patients)* | | | *Carboplatin alone (ten patients)* | | |
|---|---|---|---|---|---|---|---|---|---|
| | *Pretreatment* | *Course 1* | *Courses 2–4* | *Pretreatment* | *Course 1* | *Courses 2–4* | *Pretreatment* | *Course 1* | *Courses 2–4* |
| Number of patient courses | 6 | 6 | 10[a] | 4 | 4 | 10 | 10 | 10 | 25 |
| Creatinine clearance, $ml^{-1}$ $min^{-1}$ (median range) | 80 (68–98) | 63 (57–71) | 84 (65–93) | 78 (68–122) | 79 (65–171) | 81 (67–104) | 102 (71–143) | 106 (70–122) | 82 (62–110) |
| AUC carboplatin mg $ml^{-1}$ $min^{-1}$ (median range) | – | 9.5 (7–12) | 12.5 (8–20) | – | 9.5 (6–12) | 10 (6–13) | – | 9 (7–12) | 9 (7–13) |
| Hypotension[b], number of patient courses | – | 0 | 10 | – | 0 | 5 | – | 0 | 0 |
| Nausea ($\geq 2$)[c], number of patient courses | – | 4 | 10 | – | 4 | 7 | – | 7 | 7 |
| Lethargy ($\geq 2$)[c], number of patient courses | – | 2 | 5 | – | 0 | 1 | – | 1 | 6 |

[a]One patient had courses 3 and 4 reduced to 683 mg $m^{-2}$. [b]Decrease of systolic and/or diastolic pressure by >20%. [c]WHO grading.

**Table III** Haemoglobin, neutrophil and platelet nadir and time to platelet recovery ($> 100 \times 10^9 l^{-1}$). Median and range of all patient courses are shown

| | *Carboplatin + amifostine* | *Carboplatin* |
|---|---|---|
| *Course 1* | | |
| Hb ($g l^{-1}$) | 107 (77–136) | 98 (78–133) |
| Neutrophil ($\times 10^9 l^{-1}$) | 1.2 (0.3–13.3) | 1.4 (0.2–21.7) |
| Platelet ($\times 10^9 l^{-1}$) | 26 (14–870) | 34 (9–749) |
| Recovery time (days) | 8 (7–13) | 11.5 (9–14) |
| *Courses 2–4* | | |
| Hb ($g l^{-1}$) | 82 (69–96) | 83 (71–152) |
| Neutrophil ($\times 10^9 l^{-1}$) | 0.9 (0.3–15.6) | 1.2 (0.1–30.7) |
| Platelet ($\times 10^9 l^{-1}$) | 21 (8–515) | 23 (2–269) |
| Recovery time (days) | 13.5 (8–17) | 21.0 (20–26) |

in the CA arm had the carboplatin dose reduced or discontinued owing to pancytopenia.

Although there was no statistically significant difference in the infection incidence comparing both treatment groups (10/25 and 3/20 for C and CA respectively), patients in the C arm tended to be hospitalised more frequently mainly for i.v. antibiotics and other supportive treatment (6/25 *vs* 0/20 patient courses, $P = 0.06$).

### *Tumour response and survival*

The response rate was evaluable in 19 patients (in two patients, one in each arm, tumour size was not assessable owing to lung atelectasis); seven patients had a partial response, five in the CA arm (5/10), and two in the C arm (2/9). Four patients in the CA arm and one patient in the C arm who responded had limited disease. The median survival was 14 months and 9 months in the CA and C groups respectively.

### Discussion

Amifostine is a thiol compound that is thought to protect normal bone marrow against the toxic effects of chemotherapy while not diminishing the antineoplastic efficacy of the cytotoxic agent (Gandara *et al.*, 1990, 1991; McCulloch *et al.*, 1991; Schuchter *et al.*, 1992; Capizzi *et al.*, 1993, 1994; Treskes *et al.*, 1993). Recently published studies have shown a lessening of pancytopenia and/or a shortened time to recovery for neutrophils and platelets if amifostine was given with chemotherapy (Glover *et al.*, 1986; Glick *et al.*, 1992, 1994; Budd *et al.*, 1993; Poplin *et al.*, 1994). These therapies included agents such as cyclophosphamide (Glover *et al.*, 1986; Glick *et al.*, 1994), cisplatinum (Glover *et al.*, 1987; Mollman *et al.*, 1988; Glick *et al.*, 1992, 1994; Schiller *et al.*, 1994), mitomycin (Poplin *et al.*, 1994) and vinblastine (Poplin *et al.*, 1994). In addition, a reduction in infections requiring antibiotics and days in hospital has been reported in a large randomised phase III study of patients with ovarian cancer treated with cisplatinum and cyclophosphamide (Glick *et al.*, 1994). A possible protection of amifostine with carboplatin has been recently suggested in a phase I investigation (Budd *et al.*, 1993).

Pharmacokinetic studies in phase I clinical trials revealed that amifostine is cleared from the plasma within 6 min of the completion of a 15 min infusion (half-life time $T1/2$: $\alpha$ 0.9 min and $\beta$ 9 min) (Shaw *et al.*, 1986). These pharmacokinetic data, therefore, are important in planning clinical protection trials and support the rationale for repeated amifostine administration particularly when used with carboplatin for which the half-life is relatively long ($\alpha$; 30–60 min and $\beta$; 450–1200 min) (Van Echo *et al.*, 1989).

In the present randomised phase II study, we investigated the haematological toxicity after monotherapy with carboplatin for inoperable NSCLC. The predominant haematological toxicity associated with carboplatin is thrombocytopenia (Canetta et al., 1985). Although the severity of thrombocytopenia was not influenced by amifostine, the time to recovery appeared to be shortened compared with patients treated with carboplatin alone ($P = 0.04$) (Table III). The need for hospitalisation for i.v. antibiotic and other supportive treatment tended to be less in the amifostine group ($P = 0.06$). Although the neutrophil nadir count and time to recovery were similar, perhaps because of the analysis having been performed on the worst counts rather than the median values of the weekly counts, no other statistically significant differences were seen and the need for transfusions were similar in both patient arms. The results could also be due to the cumulative nature of carboplatin's haematological toxicity and a possible 'carry over' effect from the first course of carboplatin.

Although renal and bone marrow function were well balanced between both groups, the carboplatin AUC as back calculated from the dose given and creatinine clearances (Calvert et al., 1989) was significantly greater (7%) in the CA arm on courses 3–4. This difference is due, at least to some extent, to dose reduction of carboplatin in one patient in the C arm. This finding strengthens the results with respect to amifostine effect on platelet recovery duration and incidence of severe infection.

Amifostine led to important side-effects such as hypotension, malaise, retching and sneezing at a dose level of 910 mg $m^{-2}$ t.i.d. Subsequent doses, therefore, were reduced by 25% to 683 mg $m^{-2}$ t.i.d. At this dose level, amifostine was well tolerated and, as in other studies, nausea, vomiting, flushing, episodic sneezing, dizziness and hypotension were mild to moderate in intensity. No hypocalcaemia observed previously (Wadler et al., 1993; O'Rourke et al., 1994) was noticed and there was no evidence of cumulative toxicity from the three daily doses of amifostine.

Studies in animal models suggest an increased response rate when amifostine is administered. In mice with ovarian carcinoma xenografts (Treskes et al., 1994) amifostine administered before carboplatin had a potentiating effect on tumour growth inhibition. In a randomised trial in patients with ovarian cancer (Glick et al., 1992) complete responses and survival with a median follow-up of 40 months were similar. In the present study the number of patients is too small to allow any conclusion on response. Nevertheless, the response and survival data like the study performed in ovarian cancer (Glick et al., 1992) argues against any tumour protection by amifostine.

Taken together, no activity of amifostine on the blood cell nadir, transfusions, time to recovery of neutrophils and haemoglobin could be observed in the present study. Therefore, it was considered unreasonable to recruit additional patients onto this study. However, amifostine given with carboplatin appears to shorten the duration of carboplatin-induced thrombocytopenia and tends to reduce hospitalisation with fewer infections necessitating the use of i.v. antibiotics and less supportive care. Further randomised studies, in which carboplatin dosage is based on the AUC calculation, and in which amifostine (at a dose level of 683 mg $m^{-2}$ t.i.d.) is given with the first chemotherapy course in order to avoid a possible cumulative carboplatin toxic effect, will be needed to confirm these results.

**Acknowledgements**
We are very grateful to Mrs Linda Ashcroft for expert statistical analyses. This study was sponsored by USB Pharma. DCB is a research fellow funded by the Swiss National Science Foundation and the Bernese Cancer League.

### References

BONOMI P. (1991). Carboplatin in non-small cell lung cancer: review of the Eastern Cooperative Oncology Group trial and comparison with other carboplatin trials. *Semin. Oncol.*, **18**, 2–7.

BUDD GT, GANAPATHI R, BAUER L, MURTHY S, ADELSTEIN D, WEICK J, GIBSON V, MCLAIN D, SERGI J AND BUKOWSKI RM. (1993). Phase I study of WR-2721 and carboplatin. *Eur. J. Cancer*, **29a**, 1122–1127.

CALVERT AH, NEWELL DR, GUMBRELL LA, O'REILLY S, BURNELL M, BOXALL FE, SIDDIK ZH, JUDSON IR, GORE ME AND WILTSHAW E. (1989). Carboplatin dosage: prospective evaluation of a simple formula based on renal function. *J. Clin. Oncol.*, **7**, 1748–1756.

CANETTA R, ROZENCWEIG M AND CARTER SK. (1985). Carboplatin: the clinical spectrum to date. *Cancer Treat. Rev.*, **12** (suppl. A), 125–136.

CAPIZZI RL, SCHEFFLER BJ AND SCHEIN PS. (1993). Amifostine-mediated protection of normal bone marrow from cytotoxic chemotherapy. *Cancer*, **72**, 3495–3501.

CAPIZZI RL. (1994). Protection of normal tissues from the cytotoxic effects of chemotherapy by amifostine (ethyol): clinical experiences. *Semin. Oncol.*, **21** (suppl. 11), 8–15.

GANDARA DR, WIEBE VJ, PEREZ EA, MAKUCH RW AND DE GREGORIO MW. (1990). Cisplatin rescue therapy: experience with sodium thiosulfate, WR2721, and diethyldithiocarbamate. *Crit. Rev. Oncol. Hematol.*, **10**, 353–365.

GANDARA DR, PEREZ EA, WIEBE V AND DE GREGORIO MW. (1991). Cisplatin chemoprotection and rescue: pharmacologic modulation of toxicity. *Semin. Oncol.*, **18**, 49–55.

GLICK J, KEMP G, ROSE P, MCCULLOCH W, SCHEFFLER B AND SCHEIN P. (1992). A randomized trial of cyclophosphamide and cisplatin ± WR-2721 in the treatment of advanced epithelial ovarian cancer. *Proc. Am. Soc. Clin. Oncol.*, **11**, 109.

GLICK J, KEMP G, ROSE P, MITCHELL E, REYNOLDS R, SCHEFFLER B AND CAPIZZI R. (1994). A randomized trial of cyclophosphamide and cisplatin ± amifostine in the treatment of advanced epithelial ovarian cancer. *Proc. Am. Soc. Clin. Oncol.*, **13**, 432 (1485).

GLOVER D, GLICK JH, WEILER C, HUROWITZ S AND KLIGERMAN MM. (1986). WR-2721 protects against the hematologic toxicity of cyclophosphamide: a controlled phase II trial. *J. Clin. Oncol.*, **4**, 584–588.

GLOVER D, GLICK JH, WEILER C, FOX K AND GUERRY D. (1987). WR-2721 and high-dose cisplatin: an active combination in the treatment of metastatic melanoma. *J. Clin. Oncol.*, **5**, 574–578.

MCCULLOCH W, SCHEFFLER BJ AND SCHEIN PS. (1991). New protective agents for bone marrow in cancer therapy. *Cancer Invest.*, **9**, 279–287.

MOLLMAN JE, GLOVER DJ, HOGAN WM AND FURMAN RE. (1988). Cisplatin neuropathy. Risk factors, prognosis, and protection by WR-2721. *Cancer*, **61**, 2192–2195.

O'ROURKE N, MCCLOSKEY E AND KANIS J. (1994). WR-2721 and hypocalcemia. *J. Clin. Oncol.*, **12**, 232.

PATCHEN ML, MACVITTIE TJ AND SOUZA LM. (1992). Postirradiation treatment with granulocyte colony-stimulating factor and preirradiation WR-2721 administration synergize to enhance hemopoietic reconstitution and increase survival. *Int. J. Radiat. Oncol. Biol. Phys.*, **22**, 773–779.

POPLIN EA, LORUSSO P, LOKICH JJ, GULLO JJ, LEMING PD, SCHULZ JJ, VEACH SR, MCCULLOCH W, BAKER L AND SCHEIN P. (1994). Randomized clinical trial of mitomycin-C with or without pretreatment with WR-2721 in patients with advanced colorectal cancer. *Cancer Chemother. Pharmacol.*, **33**, 415–419.

SCHILLER JH, MEHTA M, LARSON M, STORER B, REYNOLDS R AND CAPIZZI R. (1994). Amifostine, cisplatin and vinblastine for advanced non small lung cancer. *Lung Cancer (Seventh World Conference on Lung Cancer)*, **11** (suppl. 1), 178 (A691).

SCHUCHTER LM, LUGINBUHL WE AND MEROPOL NJ. (1992). The current status of toxicity protectants in cancer therapy. *Semin. Oncol.*, **19**, 742–751.

SHAW LM, TURRISI AT, GLOVER DM, BONNER HS, NORFLEET AL, WEILER C AND KLIGERMAN MM. (1986). Human pharmacokinetics of WR-2721. *Int. J. Radiat. Oncol. Biol. Phys.*, **12**, 1501–1504.

SMITH IE. (1992). Carboplatin in small cell lung cancer: the Royal Marsden Hospital experience. *Semin. Oncol.*, **19** (1 suppl. 2), 24–27.

TRESKES M, HOLWERDA U, KLEIN I, PINEDO HM AND VAN DER VIJGH WJF. (1991). The chemical reactivity of the modulating agent WR2721 (ethiofos) and its main metabolites with the antitumor agents cisplatin and carboplatin. *Biochem. Pharmacol.*, **42,** 2125–2130.

TRESKES M AND VAN DER VIJGH WJF. (1993). WR2721 as a modulator of cisplatin- and carboplatin-induced side effects in comparison with other chemoprotective agents: a molecular approach. *Cancer Chemother. Pharmacol.*, **33,** 93–106.

TRESKES M, BOVEN E, VAN DE LOOSDRECHT AA, WIJFFELS JFAM, CLOOS J, PETERS GJ, PINEDO HM AND VAN DER VIJGH WJF. (1994). Effects of the modulating agent WR2721 on myelotoxicity and antitumour activity in carboplatin-treated mice. *Eur. J. Cancer*, **30a,** 183–187.

VAN DER VIJGH WJF AND PETERS GJ. (1994). Protection of normal tissues from the cytotoxic effects of chemotherapy and radiation by amifostine (ethyol): preclinical aspects. *Semin. Oncol.*, **21** (suppl. 11), 2–7.

VAN DER WILT CL, VAN LAAR JAM, GYERGYAY F, SMID K AND PETERS GJ. (1992). Biochemical modification of the toxicity and the anti-tumour effect of 5-fluorouracil and cis-platinum by WR-2721 in mice. *Eur. J. Cancer*, **28A,** 2017–2024.

VAN ECHO DA, EGORIN MJ AND AISN J. (1989). The pharmacology of carboplatin. *Semin. Oncol.*, **16** (suppl. 5), 1–6.

VAN LAAR JAM, VAN DER WILT CL, TRESKES M, VAN DER VIJGH WJF AND PETERS GJ. (1992). Effect of WR-2721 on the toxicity and antitumor activity of the combination of carboplatin and 5-fluorouracil. *Cancer Chemother. Pharmacol.*, **31,** 97–102.

WADLER S, HAYNES H, BEITLER JJ, GOLDBERG G, HOLLAND JF, HOCHSTER H, BRUCKNER H, MANDELLI J, SMITH H AND RUNOWICZ C. (1993). Management of hypocalcemic effects of WR2721 administered on a daily five times schedule with cisplatin and radiation therapy. *J. Clin. Oncol.*, **11,** 1517–1522.

WORLD HEALTH ORGANIZATION. (1979). *Handbook for Reporting Results of Cancer Treatment.* WHO offset publication no. 48. WHO: Geneva.

YUHAS JM, SPELLMAN JM, JORDAN SW, PARDINI MC, AFZAL SMJ AND CULO F. (1980). Treatment of tumours with the combination of WR-2721 and cis-dichlorodiammineplatinum (II) or cyclophosphamide. *Br. J. Cancer*, **42,** 574–585.